# Supplementary material for: Effects of Strain Differences, Humidity Changes, and Saliva Contamination on the Inactivation of SARS-CoV-2 by Ion Irradiation
Source: Viruses. 2024 Mar 28;16(4):520. doi: 10.3390/v16040520 (PMC11055001; doi:10.3390/v16040520)
Supplement: Supplementary file 1 [file viruses-16-00520-s001.zip › viruses-2890859-supplementary.pdf]

FigureS1

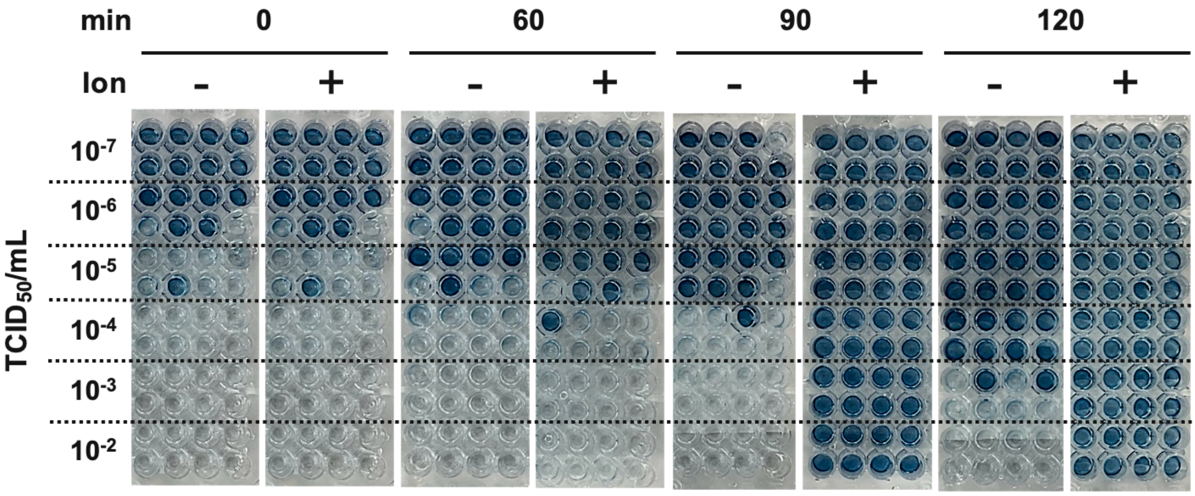

Figure S1. TCID50 per mL data pertains to the SARS-CoV-2 Omicron strain under 30% humidity conditions. "-" denotes conditions without ion exposure, while "+" signifies ion exposure. Surviving cells after viral infection were stained blue, whereas virus-infected cells remained unstained due to subsequent cell scraping. Serial dilutions of the virus solution from 10^{-1} to 10^{-6} were made. Then, 100 μL of viral solution were aliquoted to each well in quadruplicate.

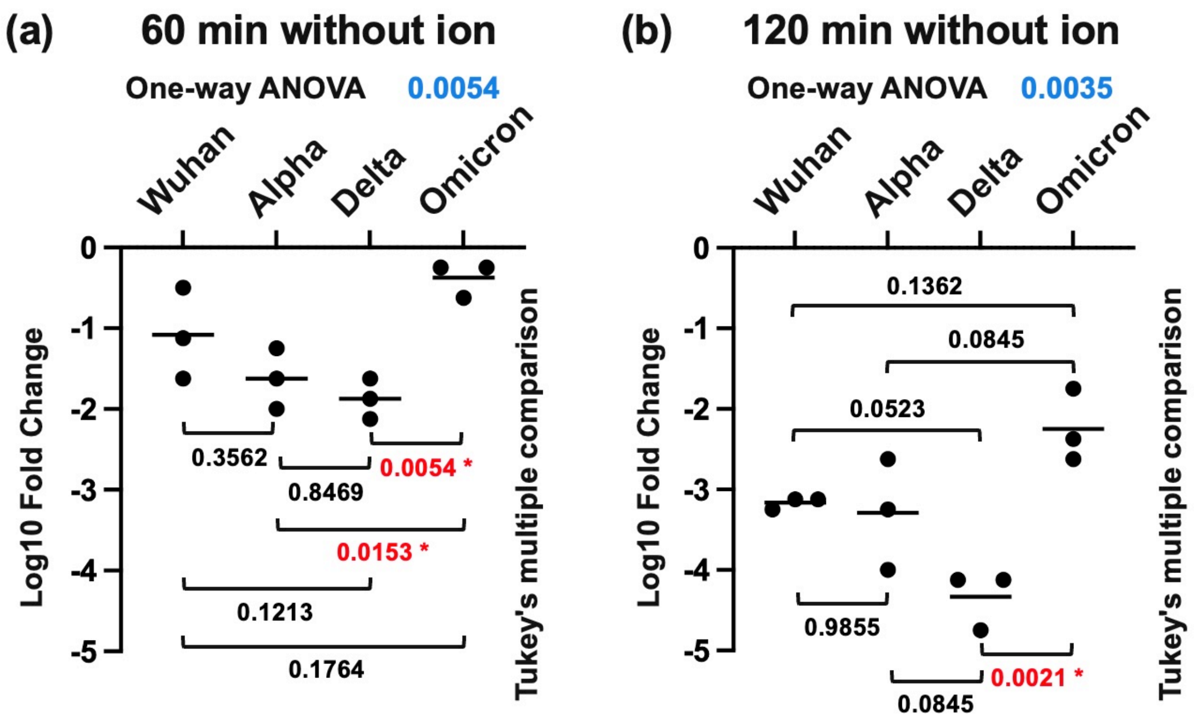

Figure S2. Inactivation of SARS-CoV-2 VOC strains in a 30% humidity environment. Four VOC strains were left in a 30% humidity either for 60-minutes **(a)** or for 120-minutes **(b)**. Then, reductions in viral $TCID_{50}$ per mL were quantified and expressed as Log10 fold change. The decrease in viral infectivity after 60 or 120 minutes was compared between the Omicron strain and other viral strains using one-way ANOVA, with the *p*-value of each comparison denoted in blue at the top. Then, statistically significant differences in the reduction of viral infectivity between each strain were evaluated using Tukey's multiple comparison test and each p-value was depicted in each graph. Values with $p < 0.05$ were highlighted in red with an asterisk.

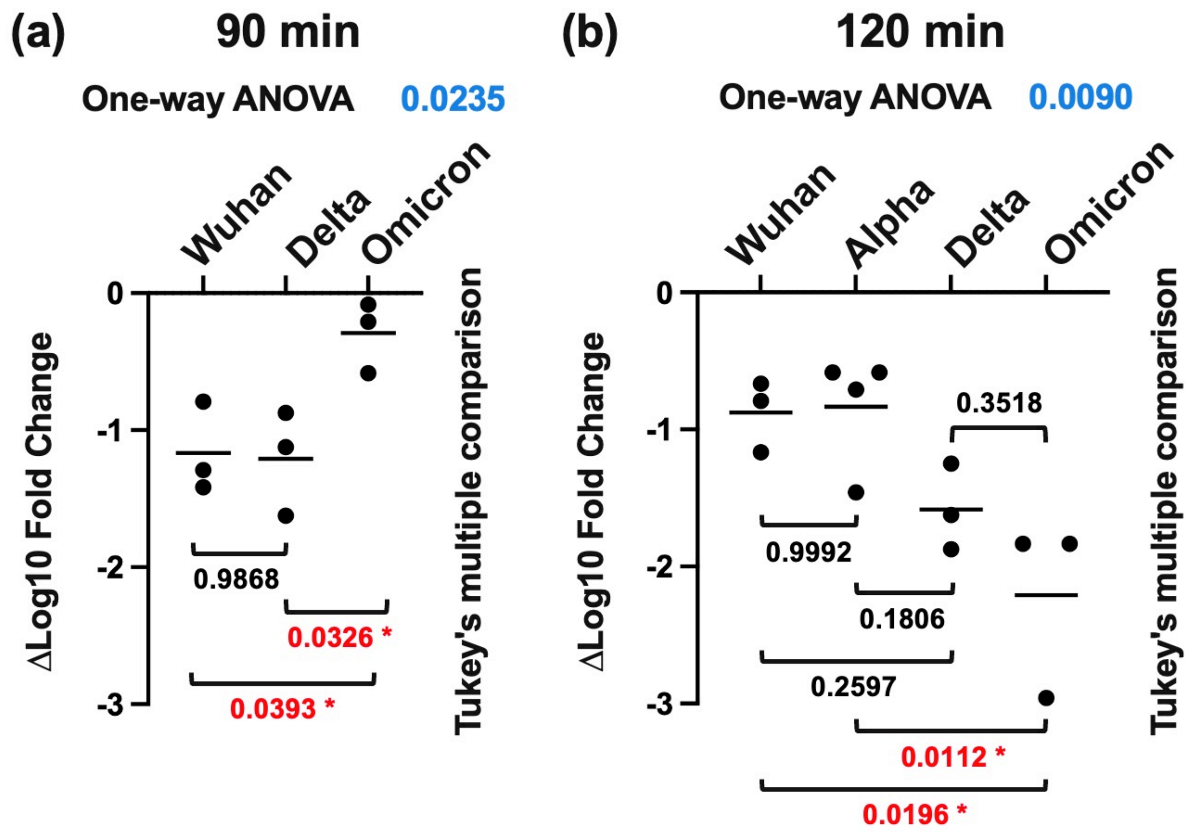

Figure S3. Inactivation of SARS-CoV-2 VOC strains in a 60% humidity environment by ion irradiation. Decrease in viral infectivity by ion irradiation in Wuhan, Delta, and Omicron strains by 90-minutes' ion irradiation **(a).** Decrease in viral infectivity by ion irradiation in Wuhan, Alpha, Delta, and Omicron strains by 120-minutes' ion irradiation **(b)**. To quantify the additional reductions in viral infectivity by ion irradiation, the difference between Log10 fold changes with and without ion irradiation was calculated, which were expressed as ΔLog10 fold changes. The effects of 90 or 120 minutes of ion irradiation on infectivity were compared across strains using one-way ANOVA, with the *p*-value of each comparison denoted in blue at the top. Following ion irradiation for either 90 minutes or 120 minutes, statistically significant differences in the reduction of viral infectivity between each strain were evaluated using Tukey's multiple comparison test, with corresponding p-value was depicted in each graph. Values with $p < 0.05$ were highlighted in red with an asterisk.

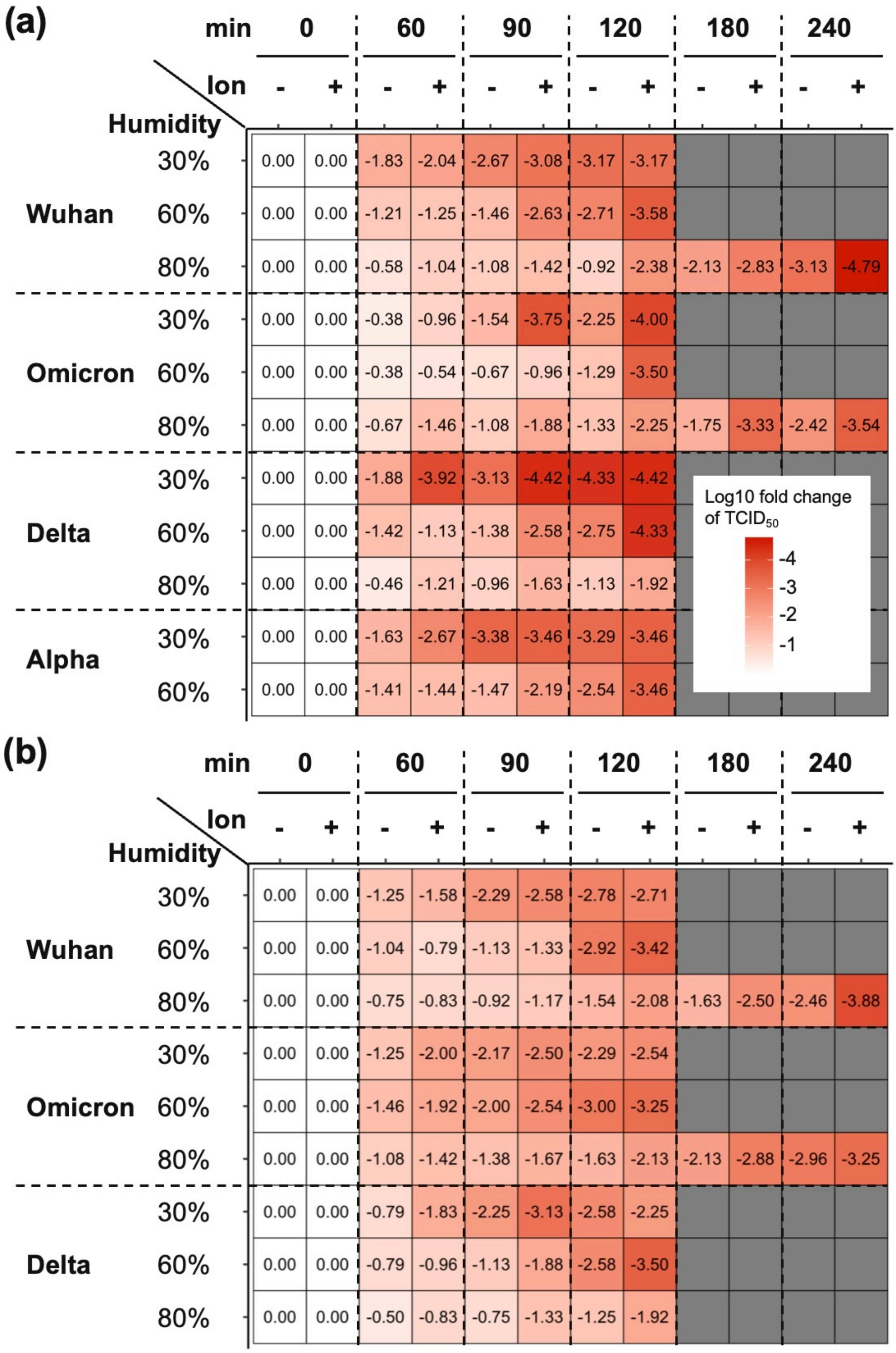

Figure S4. Heatmap representation of the mean log10 fold change of $TCID_{50}$ compared with the baseline at 0 minutes under identical humidity and ion irradiation conditions for each variant. Panel **(a)** illustrates the results in the absence of saliva, while panel **(b)** depicts observations in the presence of saliva. Each box represents the mean value of each condition, indicated by both color and number. The color scheme for the heatmap of log10-fold change is shown in the bottom right of panel **(a)**. Gray boxes denotes unassessed conditions.

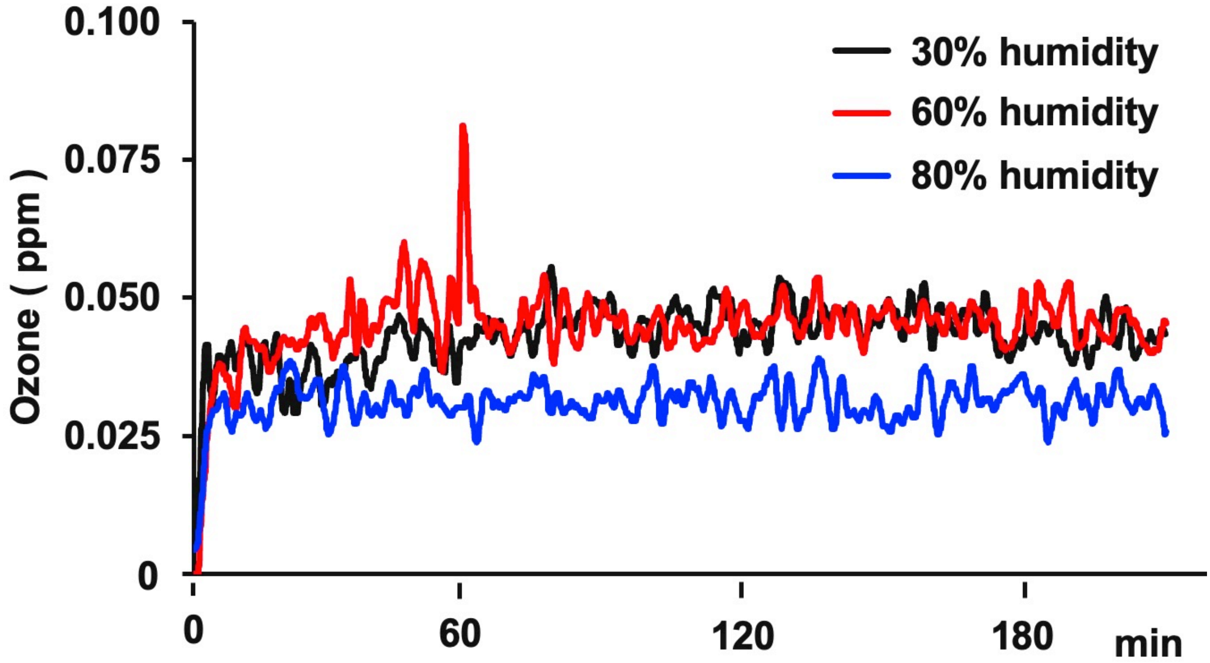

Figure S5. Changes in ozone concentration in the test box during ion irradiation experiments. Ozone concentration was measured by ozone gas analyzer (EG-700EⅢ; EBARA JITSUGYO, Tokyo, Japan).
